# Preoperative prediction of tumor budding grade in rectal cancer by combining APT histogram analysis and ADC MRI

**DOI:** 10.1186/s41747-026-00727-w

**Published:** 2026-05-20

**Authors:** Yingying Zhang, Yunxia Du, Jinghuan Huang, Ou Yang, Juntao Gong, Xiaoyue Zhang, Yun Sun, Feixiang Li, Jiaqi Wang, Gang Huang

**Affiliations:** 1 Gansu University of Chinese Medicine, Lanzhou, China; 2 Lanzhou Petrochemical General Hospital (The fourth affiliated Hospital of Gansu University of Chinese Medicine), Lanzhou, China; 3 Department of Clinical and Technical Support, Philips Healthcare, Xi’an, China; 4 Department of Radiology, Gansu Provincial Hospital, Lanzhou, China

**Keywords:** Biomarkers, Diffusion magnetic resonance imaging, Machine learning, Neoplasm staging, Rectal neoplasms

## Abstract

**Objective:**

Tumor budding (TB) is a histopathological marker of aggressive behavior and poor prognosis in rectal cancer (RC), yet not reliably evaluated preoperatively. We assessed whether histogram features from amide proton transfer-weighted (APTw) imaging and apparent diffusion coefficient (ADC) maps could serve as noninvasive biomarkers for preoperative TB grade prediction.

**Materials and methods:**

This retrospective study included 204 patients with RC from June 2023 to May 2025, divided into a training cohort (*n* = 133) and a validation cohort (*n* = 71) using a temporal split. All patients underwent preoperative APTw and diffusion-weighted imaging, and TB grade was determined histopathologically. Histogram features were extracted from whole-tumor volumes on APTw and ADC maps. Feature selection used a machine learning-based classifier, followed by univariate and multivariate logistic regression to identify independent predictors. SHapley Additive exPlanations (SHAP) were applied for interpretability, and a nomogram integrating histogram and clinical variables was constructed.

**Results:**

Five key histogram features (ADC-90%, ADC-Minimum, ADC-Range, APTw-10%, and APTw-Median) were selected. The histogram model achieved areas under the curve (AUROCs) of 0.85 (95% confidence interval [CI]: 0.79–0.92) and 0.86 (95% CI: 0.78–0.95) in the training and validation cohorts. SHAP analysis identified ADC-90% and ADC-Minimum as the most influential predictors. The combined model with histogram and clinical factors showed improved performance, with AUROCs of 0.88 (95% CI: 0.82–0.94) and 0.87 (95% CI: 0.79–0.96).

**Conclusion:**

APTw and ADC histogram features can independently predict TB grade in RC. The combined model, integrating both histogram and clinical features, further enhanced preoperative predictive accuracy.

**Relevance statement:**

This study investigates the role of imaging biomarkers in the preoperative stratification of RC, with the potential to enhance clinical decision-making and improve patient outcomes by providing more accurate, noninvasive prognostic information.

**Key Points:**

- A histogram-based model using APTw and ADC maps can predict TB grade in RC preoperatively.
- A combined model integrating clinical factors and histogram features improves predictive accuracy.
- This interpretable, contrast-free method provides a practical tool for clinicians to personalize treatment planning.

**Graphical Abstract:**

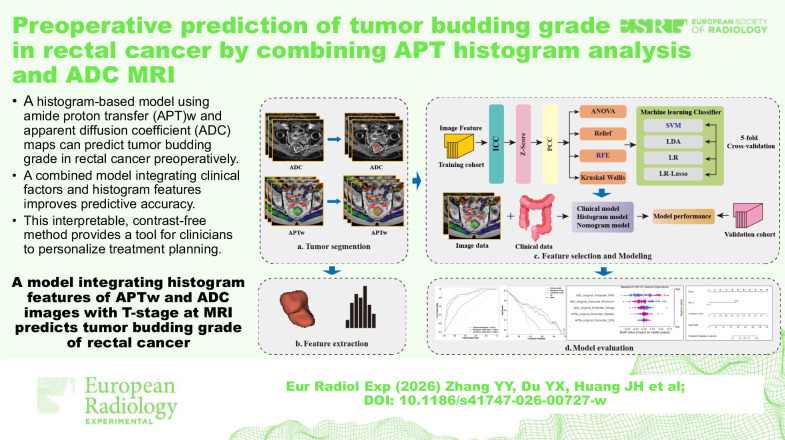

## Background

Colorectal cancer is the third most common and second most fatal malignancy worldwide, with approximately 40% of cases classified as rectal cancer (RC) [1]. The prognosis of patients with RC depends on tumor characteristics, individual patient factors, and treatment modalities [2, 3]. Tumor budding (TB) reflects a dynamic process of tumor invasion and progression [4, 5], serving as an independent risk factor associated with adverse outcomes in patients with RC. High-grade TB correlates with high tumor grade, reduced overall survival, and decreased disease-free survival [6, 7]. Moreover, it often indicates the need for adjuvant chemotherapy in patients with locally advanced RC [8–10]. Given the strong association between high-grade TB and poor prognosis, preoperatively identifying high-grade TB in patients not receiving neoadjuvant therapy could significantly influence clinical management. Understanding the risk of high-grade TB may guide decisions on more aggressive postoperative treatment, surveillance strategies, or early initiation of adjuvant chemotherapy.

Currently, TB grade is determined through pathologic examination of surgically resected specimens, with limited options for preoperative assessment in patients ineligible for surgery. Although TB grading conventionally depends on histopathology, noninvasive magnetic resonance imaging (MRI) biomarkers such as amide proton transfer-weighted (APTw) imaging and apparent diffusion coefficient (ADC) mapping can provide a more comprehensive assessment of tumor heterogeneity, serving as preoperative prognostic tools. Although previous studies have demonstrated the potential of subregional APTw histogram analysis for predicting TB in RC, these studies were often limited by small sample sizes, reliance on a single imaging sequence, and the lack of independent validation cohorts [11].

APTw imaging [12, 13] enables the noninvasive, contrast-free detection of endogenously mobile proteins and peptides within tumors, and demonstrates substantial potential for the diagnosis, grading, and prognostic evaluation of RC [14–16]. ADC is a quantitative parameter derived from diffusion-weighted imaging (DWI) that quantifies the degree of water molecule diffusion in tissues. It has been extensively used in the diagnosis and treatment of RC [17–19]. However, the average APTw signal intensity (SI) or ADC value obtained from a subjectively defined region of interest (ROI) may not accurately or comprehensively represent the overall characteristics of the tumor. This limitation contributes to inconsistencies among current studies investigating APTw and ADC in RC [15, 16, 20]. Histogram analysis based on the entire tumor contour can more accurately characterize the voxel-wise distribution of imaging signals, thereby reducing the subjectivity associated with traditional ROI delineation and enabling a quantitative assessment of intratumoral heterogeneity [21–23]. While prior studies have explored the potential of imaging biomarkers in predicting TB, none have focused specifically on the use of APTw and ADC histogram analysis for this purpose in RC.

Therefore, this study aimed to investigate the histogram features derived from APTw and ADC parameters for the preoperative prediction of TB grade in RC. The primary endpoint was to assess the ability to differentiate high-grade from low-grade TB. Secondary endpoints included evaluating the combination of imaging features and clinical factors, and model interpretability using SHapley Additive exPlanations (SHAP). We hypothesized that combining APTw and ADC histogram features would improve preoperative TB prediction accuracy, offering a biologically meaningful, noninvasive tool for personalized treatment.

## Methods

### Patients

**Fig. 1:**
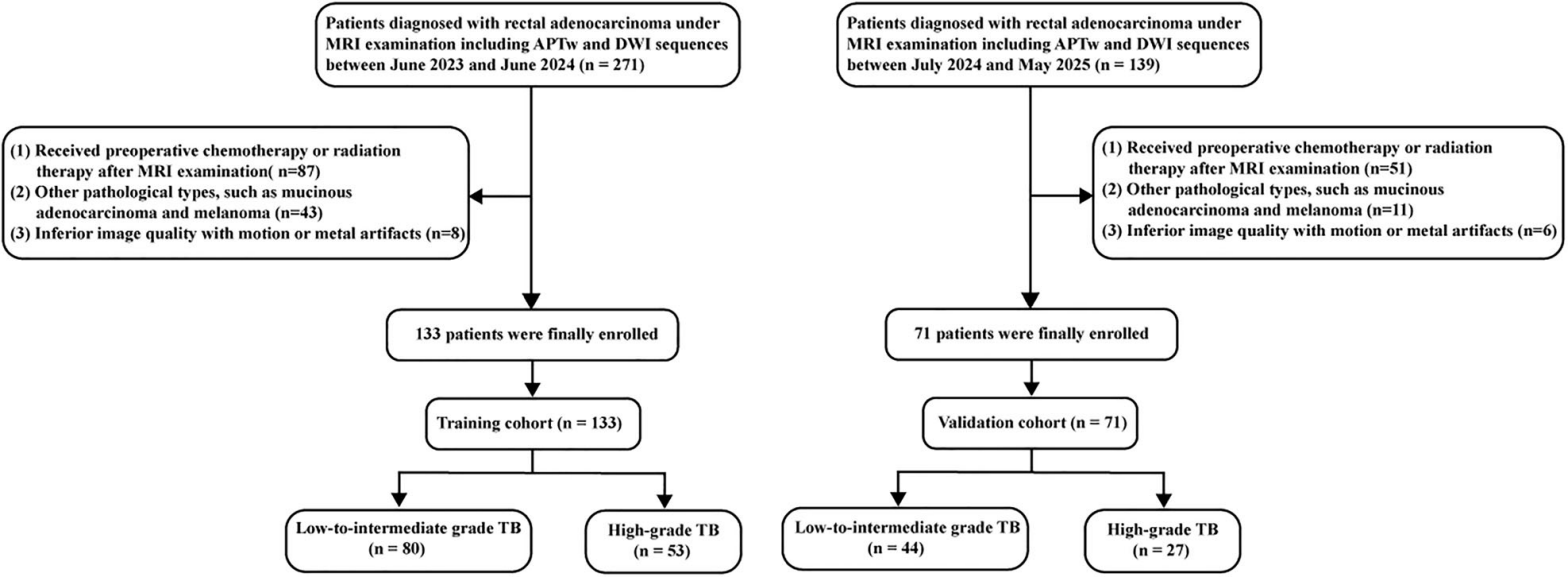
Flowchart depicting patient selection in the training and validation cohorts. APTw, Amide proton transfer-weighted; DWI, Diffusion-weighted imaging; TB, Tumor budding

This retrospective study was approved by the hospital’s ethics committee and was exempt from the requirement for informed consent. This study was conducted in accordance with the Declaration of Helsinki. We evaluated 410 consecutive patients who underwent surgical treatment for RC from June 2023 to May 2025 at our hospital. The inclusion criteria were as follows: (1) pelvic MRI performed within 2 weeks before surgery, including APTw and DWI sequences; (2) histologically confirmed rectal adenocarcinoma; and (3) comprehensive pathologic data, including TB grade. The exclusion criteria were as follows: (1) prior preoperative chemotherapy or radiotherapy (*n* = 138); (2) other pathologic types, such as mucinous adenocarcinoma and melanoma (*n* = 54); and (3) low-quality images (*n* = 14). A total of 204 patients were included, with 133 assigned to the training cohort and 71 to the validation cohort. The cohorts were split temporally within the same center, with patients enrolled between June 2023 and June 2024 forming the training cohort and those enrolled between July 2024 and May 2025 forming the validation cohort (Fig. 1). Baseline clinicopathologic data, including age, sex, histologic grade, carcinoembryonic antigen (CEA) level, and carbohydrate antigen 19-9 (CA19-9) level, were extracted from medical records. Laboratory tests for CEA and CA19-9 levels were conducted within 1 week before surgery.

### MRI protocol

MRI scans were acquired using a 3-T scanner (Ingenia Elition, Philips Healthcare) equipped with a 16-channel torso coil. Five milligrams of raceanisodamine hydrochloride was administered intramuscularly 20 min before the scan to minimize peristaltic movement. MRI scanning protocols included T1-weighted imaging, T2-weighted imaging (T2WI), high-resolution T2WI, APTw imaging, and DWI. APTw imaging is based on the chemical exchange saturation transfer process, with SI primarily reflecting the exchange of protons between bulk water and endogenous mobile proteins and peptides. Factors such as tissue pH and environmental temperature can influence APTw SI [12, 13]. ADC maps were automatically generated using a monoexponential model based on DWI SIs with *b*-values of 0 and 1000 s/mm^2^. Tumor location was assessed on axial and sagittal high-resolution T2WI.

APTw images were acquired using a three-dimensional (3D) turbo spin-echo (TSE)-Dixon pulse sequence with chemical shift-selective fat suppression. The acquisition time for each sequence was approximately 5 min, with APTw using a 2-second radiofrequency saturation pulse train. The APTw images were computed using 7 saturation frequency offsets (± 3.5, ± 3.42, ± 3.58, and ‒1.56 ppm). A B_0_ map for the asymmetry calculation was derived from 3 acquisitions at + 3.5 ppm using the mDIXON algorithm. The 3D APTw imaging parameters were as follows: repetition time/echo time = 6,458/8.8 ms; flip angle = 90°; matrix = 156 × 154; slice thickness = 6 mm; field of view = 280 × 260 mm^2^; and spatial resolution = 1.8 × 1.8 × 6 mm^3^. The detailed MRI sequence parameters are presented in Supplementary File 1 and Table S1.

### MRI characteristic evaluation

The MRI images were reviewed independently by two experienced radiologists (each with more than 8 years of experience), blinded to clinical and pathologic data. The T stage (MRI-T) was determined by evaluating the depth of tumor invasion into the intestinal wall and its involvement with the rectal mesentery and surrounding organs. The N stage (MRI-N) was assessed based on lymph node morphology, border characteristics, and SI. In cases of disagreement, the two radiologists reached a consensus through discussion.

### Histogram feature extraction

**Fig. 2:**
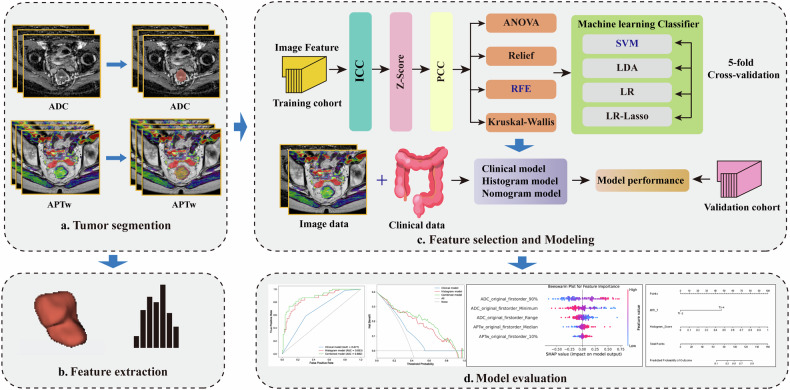
Flowchart illustrating the study design. ADC, Apparent diffusion coefficient; APTw, Amide proton transfer-weighted; LDA, Linear discriminant analysis; LR, Logistic regression; LR-Lasso, Lasso logistic regression; PCC, Pearson correlation coefficient; SVM, Support vector machine

The APTw source images were transferred to the IntelliSpace Portal workstation (Philips Healthcare) for postprocessing to generate quantitative APTw maps. Pseudocolor displays were created for visualization only to facilitate tumor delineation. The quantitative APTw maps and ADC maps were exported in Digital Imaging and Communications in Medicine (DICOM format and imported into 3D Slicer (version 5.4.0, https://www.slicer.org) for tumor segmentation. First, APTw and ADC images were aligned using rigid registration in 3D Slicer, with cross-sectional T2WI images overlaid solely for anatomical reference. Next, a volume of interest (VOI) was manually delineated layer by layer along the tumor margin on the fused image, excluding fluid, gas, and intestinal lumen contents. After segmentation on APTw, the same volumes of interest were propagated to the ADC images. Subsequently, the histogram features were extracted using PyRadiomics (v.1.3.0, http://github.com/Radiomics/pyradiomics) based on Python (v. 3.7.1, http://www.python.org), including minimum, maximum, mean, 10th percentile, 90th percentile, interquartile range, median, range, mean absolute deviation, robust mean absolute deviation, root mean square, energy, total energy, entropy, kurtosis, skewness, uniformity, and variance. The complete PyRadiomics parameter configuration file (YAML) used for radiomics feature extraction is provided as Supplementary File 2. The VOI segmentation was performed by Radiologist 1 (with 5 years of experience in abdominal imaging), who was blinded to the pathological findings. To assess intraobserver reproducibility, Radiologist 1 performed a second VOI segmentation on 50 randomly selected patients one month later. For interobserver variability, VOI segmentation was independently performed by Radiologist 2 (with 8 years of experience in abdominal imaging). Intraobserver and interobserver reliability were both assessed using the intraclass correlation coefficient (ICC) with a two-way random effects model and absolute agreement (ICC(2,1)), with results indicating good agreement (ICC > 0.75). The tumor segmentation and feature selection process is illustrated in Fig. 2.

### Histogram feature selection

All histogram features were imported into the radiomics-based open-source software FeAture Explorer (version 0.5.12; https://github.com/salan668/FAE). The histogram features were first *Z*-score normalized, and feature dimensionality was reduced using Pearson correlation coefficients with a threshold of 0.9. Further feature selection was performed using analysis of variance, relief algorithm, recursive feature elimination, and Kruskal–Wallis test. All preprocessing steps, including *Z*-score normalization, were performed within each training fold of the 5-fold cross-validation to avoid information leakage from the test folds or the held-out temporal validation cohort. Feature selection and hyperparameter optimization were then carried out exclusively within the training folds. Multiple machine learning classifiers, including support vector machine, linear discriminant analysis, logistic regression, and Lasso logistic regression, were evaluated using cross-validation on the training data. To address the class imbalance, oversampling was applied to the minority class (high-grade TB) during model training. Synthetic samples were generated using the SMOTE algorithm to balance the class distribution. Then, the validation dataset was completely held out from feature selection and model training and was used solely for final performance evaluation. Finally, the histogram features identified by the optimally performing classifier were used to build the model, and the histogram score was calculated using a linear combination.

### Modeling and evaluation of effectiveness

In the training cohort, the independent risk factors for TB grade were identified using univariate and multivariate logistic regression analyses. Relevant clinicopathologic features were then selected to construct the clinical model. A combined model was developed by integrating the histogram score with these selected clinical features. The diagnostic performance of each model was evaluated using the area under the receiver operating characteristic curve (AUROC) and compared using the DeLong test. The sensitivity, specificity, and accuracy were also calculated. The optimal operating point was selected based on the highest Youden’s Index. Additionally, a nomogram was generated to provide a visual representation of the model, and decision curve analysis was performed to evaluate its clinical applicability. To further interpret the contributions of individual histogram features to the model’s predictions, SHAP analysis was performed, which quantified the relevance of each feature and illustrated how specific histogram characteristics influenced the predicted TB grade. This approach enhanced both the interpretability and clinical relevance of the combined model.

### Histopathologic analysis

TB was assessed by two experienced pathologists (a board-certified pathologist with three years of diagnostic experience and a senior pathologist with eight years of diagnostic experience) according to the International TB Consensus Conference 2016 guidelines [4]. Hematoxylin and eosin-stained sections were first scanned at medium magnification (10×) to identify areas with the highest density of tumor buds along the invasive front, followed by evaluation at 20× magnification. Bud counts were normalized to represent the number of tumor buds per 0.785 mm². TB was classified based on bud counts as follows: Bd 1 (low grade), 0‒4 buds; Bd 2 (intermediate grade), 5‒9 buds; and Bd 3 (high grade), 10 or more buds. For clinical relevance, the pathology endpoint was dichotomized into low-to-intermediate-grade (Bd 1 + 2) and high-grade (Bd 3) groups, as high-grade TB is associated with poorer prognosis and higher recurrence risk, providing meaningful stratification in RC.

### Statistical analysis

Interobserver and intraobserver reliability were assessed using the ICC, with values ≥ 0.75 indicating acceptable reliability. The normality of continuous variables was assessed using the Kolmogorov–Smirnov test. Normally distributed continuous variables were compared using independent-samples *t* tests and presented as mean ± standard deviation. In contrast, non-normally distributed variables were assessed using the Mann–Whitney *U*-test and presented as median (Q1, Q3). Categorical variables were compared using chi-square or Fisher’s exact tests and expressed as percentages. Univariate and multivariate logistic regression analyses were performed to identify factors strongly associated with TB grade. Confidence intervals (CIs) for model performance metrics were estimated using bootstrap resampling. The statistical analysis of the data was conducted using R (version 4.3.0), SPSS (version 25.0), and Python (version 3.7.1) software. All tests were 2-sided, and a *p*-value < 0.05 was considered statistically significant.

## Results

### Comparison of clinicopathologic characteristics of patients between various TB grades

The study included 133 patients in the training cohort and 71 patients in the validation cohort. Among the 133 patients with RC, 80 had low-to-intermediate-grade TB, and 53 had high-grade TB; in the validation cohort, 44 patients were in the low-to-intermediate-grade group and 27 in the high-grade group. The clinicopathologic characteristics of patients in both cohorts are summarized and compared in Table 1. No significant differences were observed between the training and validation cohorts with respect to key clinical characteristics. In the training cohort, significant differences in MRI-T, histologic grade, and CEA (*p* = 0.002, 0.048, and 0.002; respectively) were observed. No significant differences in age (*p* = 0.462), sex (*p* = 0.462), MRI-N stage (*p* = 0.811), or CA19-9 (*p* = 0.470) were observed between the TB grades. In contrast, no significant differences were observed in the validation cohort because of the small sample size.

**Table 1:** Comparison of clinicopathologic characteristics of patients in the training and validation cohorts.

Characteristic	Training cohort (*n* = 133)			Validation cohort (*n* = 71)			*p-*value^#^
	Low-to-intermediate (*n* = 80)	High (*n* = 53)	*p-*value^*^	Low-to-intermediate (*n* = 44)	High (*n* = 27)	*p-*value^*^	
Sex, *n* (%)			0.462			0.259	0.111
Male	51 (63.7)	30 (56.6)		24 (54.5)	11 (40.7)		
Female	29 (36.3)	23 (43.4)		20 (45.5)	16 (59.3)		
Age (year), median (Q1, Q3)	64 (58, 72)	65 (55, 72)	0.462	64 (57.8, 68.3)	60 (54, 69.5)	0.402	0.082
MRI-T stage, *n* (%)			0.002			0.360	0.794
T1-2	16 (20)	1 (1.9)		8 (18.2)	2 (7.4)		
T3-4	64 (80)	52 (98.1)		36 (81.8)	25 (92.6)		
MRI-N stage, *n* (%)			0.811			1.000	0.182
N0	18 (22.5)	11 (20.8)		6 (13.6)	4 (14.8)		
N1-2	62 (77.5)	42 (79.2)		38 (86.4)	23 (85.2)		
Histologic grade, *n* (%)			0.048			1.000	0.169
Well/moderate	80 (100.0)	49 (92.5)		40 (90.9)	25 (92.6)		
Poor	0 (0.0)	4 (7.5)		4 (9.1)	2 (7.4)		
CEA level, *n* (%)			0.002			0.164	0.12
< 5 ng/mL	55 (68.7)	22 (41.5)		33 (75.0)	16 (59.3)		
≥ 5 ng/mL	25 (31.3)	31 (58.5)		11 (25.0)	11 (40.7)		
CA19-9 level, *n* (%)			0.470			0.830	0.817
< 37 U/mL	70 (87.5)	44 (83.0)		38 (86.4)	22 (81.5)		
≥ 37 U/mL	10 (12.5)	9 (17.0)		6 (13.6)	5 (18.5)		

CA19-*9* Carbohydrate antigen 19-9, *CEA* Carcinoembryonic antigen, *MRI-N* Magnetic resonance imaging N staging, *MRI-T* Magnetic resonance imaging T staging

Normal range of CEA: 0–5.00 ng/mL; normal range of CA19-9: 0–37.00 U/mL.

^*^Comparison between the low and high-to-intermediate group

^#^Comparison between training cohort and validation cohort

### Performance of the clinical model

Univariate analysis identified MRI-T and CEA as substantial risk factors for TB grade (Table 2). Multivariate analysis of the clinical model confirmed that MRI-T (odds ratio [OR] 9.13; 95% CI: 1.14‒73.01; *p* = 0.037) and CEA (OR 2.42; 95% CI: 1.15‒5.09; *p* = 0.020) were associated with TB grade (Table 3). For TB grade prediction, the clinical model yielded an AUROC of 0.68 (95% CI: 0.60‒0.76), with a sensitivity of 70% and a specificity of 59% in the training cohort. In the validation cohort, the model yielded an AUROC of 0.61 (95% CI: 0.50‒0.74), with a sensitivity and specificity of 77% and 41%, respectively (Table 4).

**Table 2:** Univariate analysis of factors associated with TB grade in RC

Risk factor	*b*-value	Standard error	*Z*	OR	95% CI	*p*-value
MRI-T						
T1–2	Reference	–	–	–	–	–
T3–4	2.56	1.05	2.45	13.00	1.67–101.30	0.014
CEA						
< 5 ng/L	Reference	–	–	–	–	–
≥ 5 ng/L	1.13	0.37	3.07	3.10	1.51–6.38	0.002
Histogram_score	1.14	0.21	5.34	3.11	2.05–4.72	< 0.001

“–” means this item is not applicable

*CEA* Carcinoembryonic antigen, *CI* Confidence interval, *MRI-T* Magnetic resonance imaging T staging, *RC* Rectal cancer, TB Tumor budding

**Table 3:** Multivariate analysis of factors associated with TB grade in RC

Risk factor	Clinical model		Combined model	
	Odds ratio (95% CI)	*p*-value	Odds ratio (95% CI)	*p*-value
MRI-T	9.13 (1.14–73.01)	0.037	13.84 (1.50–128.01)	0.021
CEA	2.42 (1.15–5.09)	0.020		
Histogram_score			3.37 (2.09–5.44)	< 0.001

*CEA* Carcinoembryonic antigen, *CI* Confidence interval, *MRI-T* Magnetic resonance imaging T staging, *RC* Rectal cancer, TB Tumor budding

**Table 4:** Predictive performance of the clinical, histogram, and combined models

Model	AUROC (95% CI)	Accuracy (95% CI)	Sensitivity (95% CI)	Specificity (95% CI)	PPV (95% CI)	NPV (95% CI)
Clinical						
Training cohort	0.68 (0.60–0.76)	0.65 (0.57–0.73)	0.70 (0.60–0.80)	0.59 (0.45–0.72)	0.72 (0.62–0.82)	0.56 (0.43–0.70)
Validation cohort	0.61 (0.50–0.74)	0.63 (0.51–0.75)	0.77 (0.65–0.90)	0.41 (0.22–0.60)	0.68 (0.55–0.81)	0.52 (0.31–0.74)
Histogram						
Training cohort	0.85 (0.79–0.92)	0.79 (0.71–0.86)	0.76 (0.67–0.86)	0.83 (0.73–0.93)	0.87 (0.79–0.95)	0.70 (0.59–0.81)
Validation cohort	0.86 (0.78–0.95)	0.80 (0.61–0.89)	0.80 (0.68–0.92)	0.82 (0.67–0.96)	0.88 (0.77–0.98)	0.71 (0.55–0.87)
Combined						
Training cohort	0.88 (0.82–0.94)	0.84 (0.77–0.90)	0.91 (0.85–0.97)	0.74 (0.62–0.86)	0.84 (0.76–0.92)	0.85 (0.74–0.95)
Validation cohort	0.87 (0.79–0.96)	0.80 (0.69–0.89)	0.77 (0.65–0.90)	0.85 (0.72–0.99)	0.90 (0.80–0.99)	0.70 (0.54–0.85)

*AUROC* Area under the receiver operating characteristic curve, *CI* Confidence interval, *NPV* Negative predictive value, *PPV* Positive predictive value

### Performance of histogram parameters

**Fig. 3:**
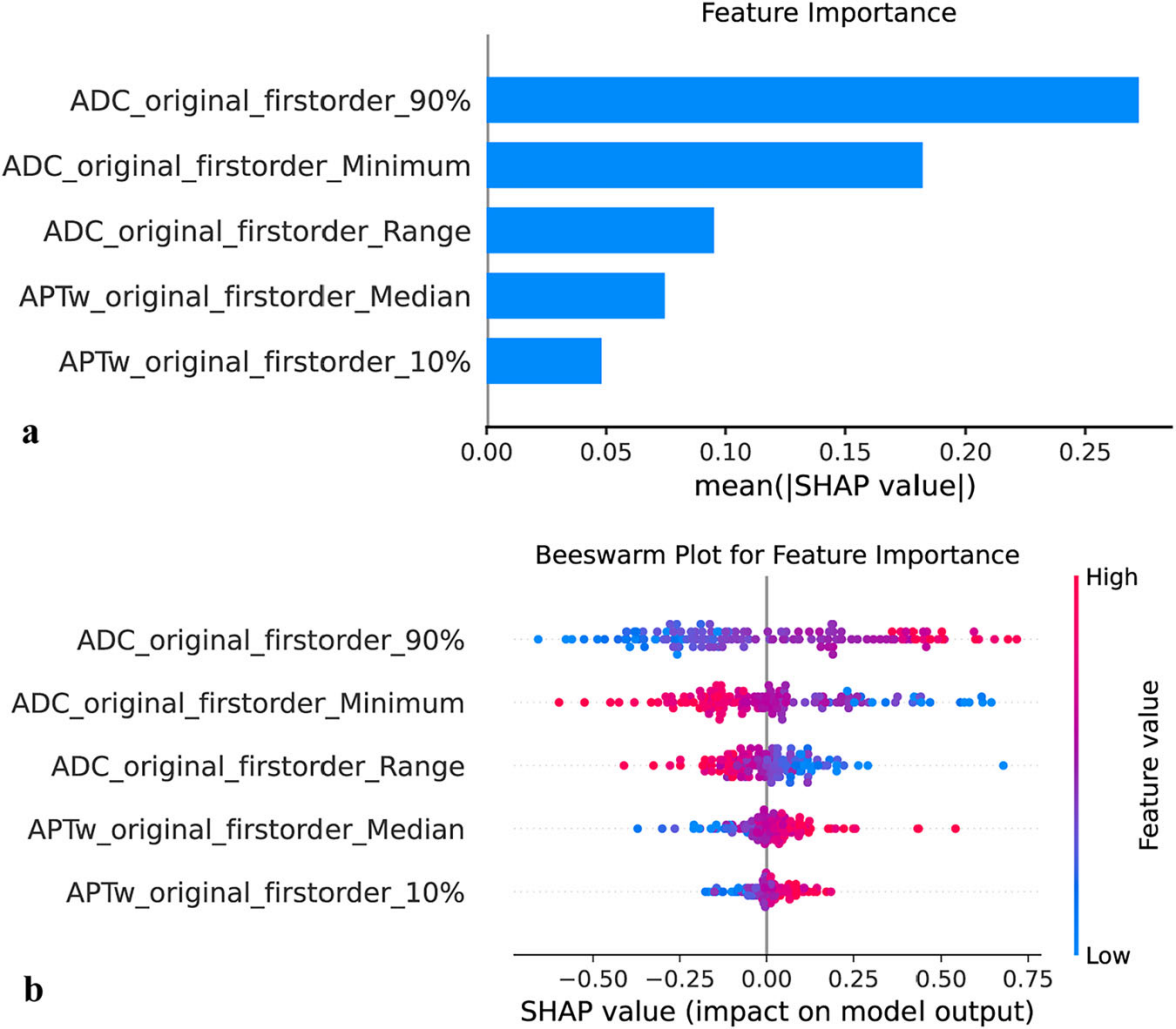
SHAP summary plots of the histogram model. **a** Ranking of feature contribution in the final model output. **b** Distribution of SHAP values for each feature, illustrating their contribution to the model prediction. Red and blue indicate higher and lower feature values, respectively, with their position on the *x*-axis reflecting the direction and magnitude of impact on the outcome. SHAP, SHapley Additive exPlanations; ADC, Apparent diffusion coefficient; APTw, Amide proton transfer-weighted

For histogram analysis, 18 features were extracted from the VOIs of both the APTw and ADC sequences, yielding a total of 36 features. Reproducibility of the extracted histogram features was assessed using ICC analysis. Intraobserver ICC values for all features were greater than 0.75. Interobserver ICC results also demonstrated good reproducibility, with detailed results provided in Supplementary File 1 and Table S2. Using an optimal support vector machine learning algorithm, 5 histogram features with nonzero coefficients were selected (Supplementary File 1 and Table S3). The differences among these 5 optimal feature groups are displayed in Supplementary File 1 and Fig. S1. These features were used to construct a histogram model and calculate the histogram score. The histogram model achieved an AUROC of 0.85 (95% CI: 0.79‒0.92) in the training set, with a sensitivity of 76% and a specificity of 83%. In the validation set, the model yielded an AUROC of 0.86 (95% CI: 0.78‒0.95), with a sensitivity of 80% and a specificity of 82% (Table 4). The SHAP analysis identified (Fig. 3a) ADC-90% and ADC-Minimum as the most influential predictors. The SHAP beeswarm plot (Fig. 3b) further illustrated that higher ADC-90% values and lower ADC-Minimum values were associated with an increased probability of high-grade TB, whereas greater APTw-10% and elevated APTw-Median also contributed positively to the prediction.

### Performance of the combined model

**Fig. 4:**
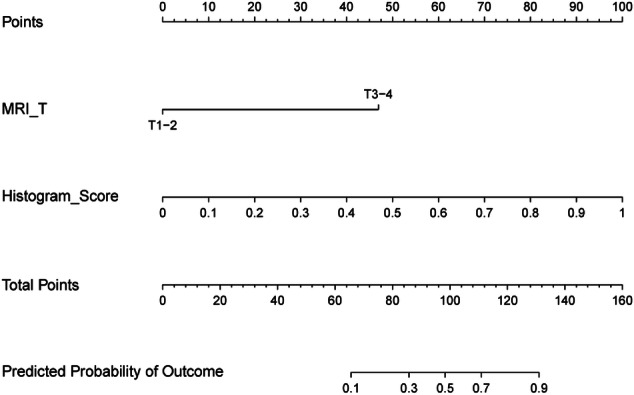
Nomogram of the combined model for TB grading in RC. MRI-T, Magnetic resonance imaging T staging

**Fig. 5:**
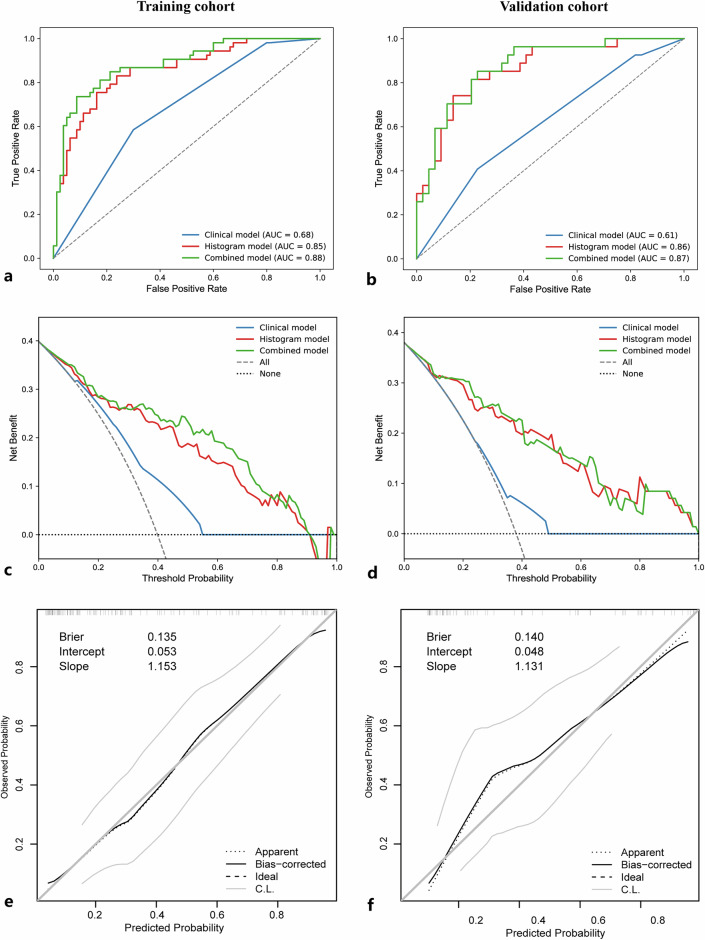
Model evaluation with ROC analysis, decision curves, and calibration in the training and validation cohorts. **a**, **b** ROC curves for the clinical, histogram, and combined models in the training and validation datasets, respectively. Decision curves indicate the clinical net benefit for the training (**c**) and validation (**d**) datasets. Calibration curves of the combined model demonstrating the agreement between predicted probabilities and observed outcomes in the training (**e**) and validation (**f**) cohorts. AUROC, Area under the ROC curve; ROC, Receiver operating characteristic

The multivariate analysis results for the combined model are summarized in Table 3. The MRI-T (OR 13.84; 95% CI: 1.50‒128.02; *p* = 0.021) and histogram score (OR 3.37; 95% CI: 2.09‒5.44; *p* < 0.001) were identified as independent predictors of TB grade. A combined model incorporating MRI-T and histogram score was developed to predict TB grade, and a nomogram was subsequently constructed to visualize the model (Fig. 4). The AUROC value, accuracy, sensitivity, and specificity of each model are presented in Table 4. The combined model demonstrated the highest diagnostic performance, achieving an AUROC of 0.88 (95% CI: 0.82‒0.94) in the training cohort and an AUROC of 0.87 (95% CI: 0.79–0.96) in the validation cohort (Fig. 5a, b).

### Comparative analysis of model performance

**Fig. 6:**
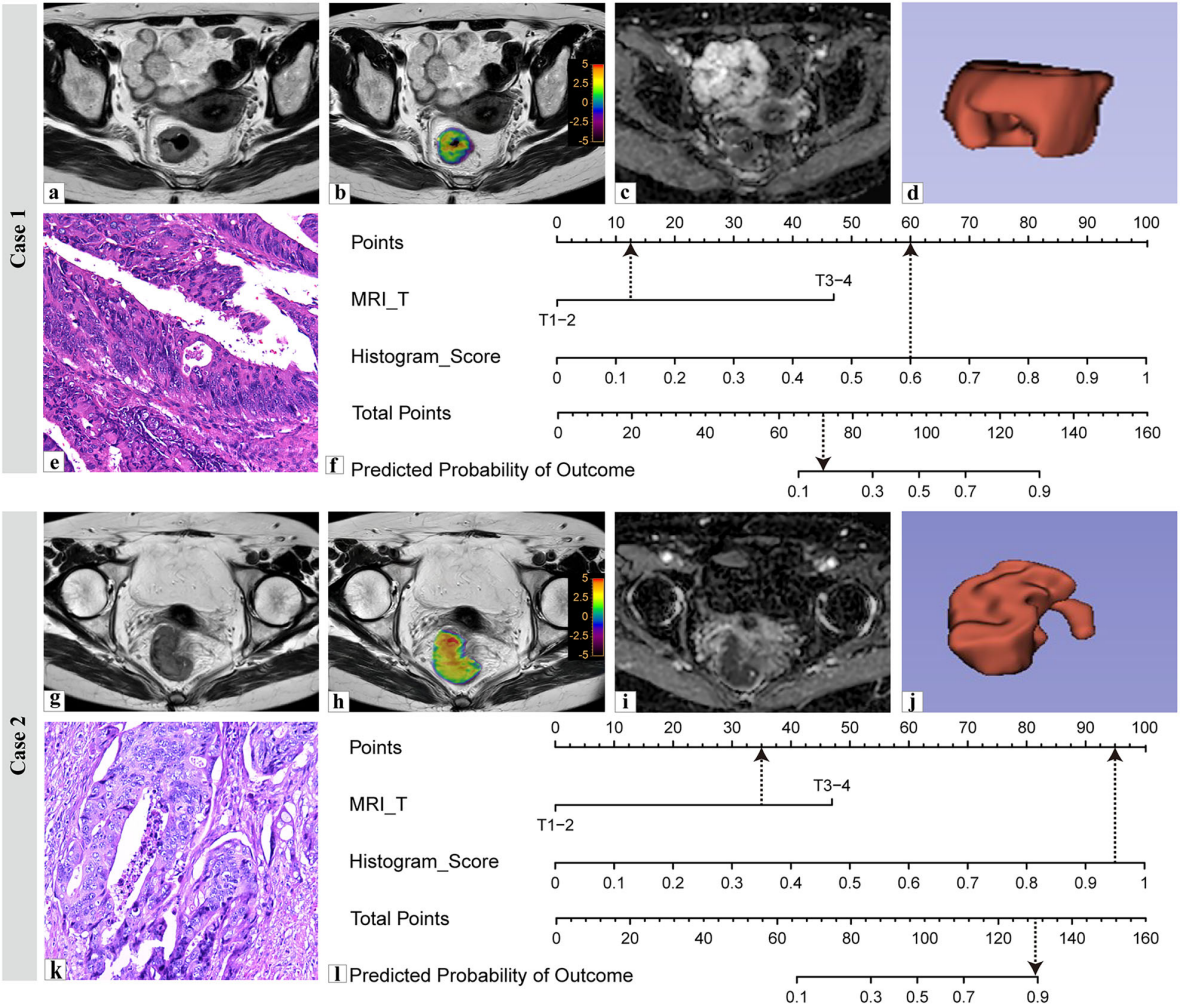
Nomogram method for calculating TB grade to predict the risk score of patients with RC. **a**–**f** Images from a 57-year-old woman with low-grade TB: (**a**) axial T2WI revealing diffuse thickening of the intestinal wall with a low signal; (**b**) APTw image fused with T2WI; (**c**) ADC image; (**d**) 3D VOI; (**e**) pathology image; and (**f**) nomogram indicating a risk score of 72, predicting a high-grade TB risk of 0.17. **g**–**l** Images from a 68-year-old woman with high-grade TB: (**g**) axial T2WI revealing diffuse thickening with low signal; (**h**) APTw image fused with T2WI; (**i**) ADC image; (**j**) 3D VOI; (**k**) pathology image; and (**l**) nomogram with risk score of 130, predicting a high-grade TB risk of 0.89. 3D, Three-dimensional; ADC, Apparent diffusion coefficient; APTw, Amide proton transfer-weighted; RC, Rectal cancer; ROI, Region of interest; TB, Tumor budding; T2WI, T2-weighted imaging

According to the DeLong test, the combined model demonstrated higher diagnostic performance than the histogram model (*p* = 0.029) and the clinical model (*p* < 0.001) in the training cohort. However, in the validation cohort, the difference was not significant compared with the histogram model (*p* = 0.244) but remained significant compared with the clinical model (*p* < 0.001). The decision curve analysis indicated that the combined model provided a higher clinical net benefit than “treat all” or “treat none” strategies when the threshold probability ranged between 0.38 and 0.40 in both the training and validation cohorts (Fig. 5c, d). The calibration curves confirmed the predictive accuracy of the combined model (Fig. 5e, f). The Hosmer–Lemeshow test yielded nonsignificant results in the training and validation cohorts, suggesting no significant lack of fit. (*p* = 0.402 and 0.487, respectively). In the training cohort, the calibration intercept and slope were 0.053 and 1.153, with a Brier score of 0.135. In the validation cohort, the intercept and slope were 0.048 and 1.131, with a Brier score of 0.140, further supporting the model’s calibration. Figure 6 illustrates representative cases of low-to-intermediate-grade and high-grade TB in RC.

## Discussion

This study developed and validated a preoperative model for TB grading in RC using whole-lesion histogram features derived from APTw and ADC images. The combined model, integrating MRI-T and histogram scores, demonstrated the highest performance (AUROCs 0.88 and 0.87 in the training and validation cohorts, respectively), offering a noninvasive approach for TB risk assessment and supporting individualized treatment planning.

Whole-tumor APTw histogram analysis has been reported to enhance the characterization of features such as extramural venous invasion in rectal adenocarcinoma [24]. In our study, the histogram model, which outperformed the clinical model, revealed that patients with high-grade TB exhibited higher ADC-90% and lower ADC-Minimum, consistent with the coexistence of necrotic/cystic regions and densely cellular invasive fronts [25]. Similar results, with higher ADC percentiles observed in more aggressive lesions, have also been reported in other oncologic settings [26–28]. Furthermore, high-grade TB demonstrated significantly increased APTw-10% and APTw-Median values. The APTw-10% parameter represents the 10th percentile of the APTw SI distribution and reflects the lower bound of APT values, whereas APTw-Median denotes the median APT SI, capturing the central tendency of the distribution. An elevation of APTw-10% indicates an upward shift in the overall APT signal profile and a reduction in low-APT regions, indicating globally increased intracellular protein content. This finding aligns with the heightened cellular proliferation characteristic of high-grade TB [29]. SHAP analysis further elucidated the model’s decision-making process by confirming that ADC-90% and ADC-Minimum were the most influential predictors, followed by ADC-Range, APTw-Median, and APTw-10%. The SHAP beeswarm plot demonstrated that higher ADC-90% and APTw-Median, along with lower ADC-Minimum and larger ADC-Range, were strongly correlated with high-grade TB. These findings indicate that the model’s predictions were driven by biologically meaningful imaging features rather than noninterpretable “black-box” mechanisms.

High-grade tumors often exhibit elevated APTw SI, reflecting abnormal protein synthesis and proliferative activity [30–32]. Prior studies have associated higher APTw SI with reduced differentiation, advanced stage, nodal metastasis, and positive extramural vascular invasion [15]. Similarly, several studies have demonstrated higher APTw SI in high-grade TB, potentially related to epithelial–mesenchymal transition. Epithelial–mesenchymal transition enhances invasiveness in tumor epithelial cells, accelerates membrane–macromolecule exchange, and promotes release and accumulation of proteins and peptides, thereby elevating APTw SI [5, 33]. Moreover, high-grade TB cells typically proliferate more rapidly and have higher intracellular concentrations of mobile proteins and polypeptides, further increasing APTw SI. Neovascularization at the invasive front of high-grade tumors may also contribute to elevated hemoglobin and plasma protein content, enhancing local APTw SI [34]. Conversely, lower ADC values in high-grade TB likely reflect increased cellular density and more restricted water diffusion at the invasive tumor front, consistent with previous findings [35]. While epithelial–mesenchymal transition and neovascularization are plausible mechanisms that could explain the elevated APTw SI in high-grade TB, these mechanisms require further experimental validation in future studies. In our cohort, mean APTw and ADC values revealed no significant differences between TB subgroups, underscoring the limitation of simple average measurements in capturing microregional heterogeneity. Histogram-based descriptors provided a more sensitive assessment by incorporating information from the entire distribution. These results demonstrate that the model’s decision-making process was driven by biologically plausible imaging features, thereby enhancing both its interpretability and clinical relevance.

TB is an indicator of tumor invasiveness and is often associated with a more advanced tumor stage [36]. Consistent with prior literature, high-grade TB in our study correlated predominantly with advanced T stage and a higher incidence of preoperative CEA positivity, suggesting that aggressive biology may be reflected by both imaging and serum markers. The clinical model based on MRI-T and CEA alone demonstrated only modest predictive ability (AUROCs 0.667 and 0.614 in the training and validation cohorts, respectively), highlighting the added value of imaging heterogeneity descriptors. It is noteworthy that the OR for MRI T stage (T3–4 *versus* T1–2) showed a wide 95% CI in the univariate analysis. This is likely attributable to the relatively small number of early-stage (T1–2) tumors and the strong association between advanced T stage and high-grade TB, which can lead to unstable estimates and inflated CIs in logistic regression.

The combined model, presented as a nomogram, offered a user-friendly tool for clinical application. Accurate preoperative stratification of TB grade may assist in optimizing surgical decision-making and identifying patients who may benefit from intensified neoadjuvant or adjuvant therapy. By providing early identification of high-grade TB, the model could guide clinicians in selecting more aggressive treatment strategies, including adjusting the surgical approach, increasing the frequency of postoperative monitoring, and initiating timely adjuvant therapies. This model could ultimately help reduce unnecessary treatments for low-risk patients, improve personalized care, and enhance overall clinical efficiency.

This study has several limitations. First, external validation in independent, multicenter cohorts and prospective studies, as well as multi-vendor validation, are necessary before clinical implementation. Second, its retrospective design and relatively small sample size may have introduced selection bias and increased the risk of model overfitting. Third, only histogram-based features of APTw and ADC images were analyzed; other radiomic feature classes (*e.g.,* texture, wavelet, or higher-order features) were not explored and could further improve performance. Fourth, the exclusion of patients who had received any form of chemotherapy or radiotherapy (138/410) is a significant limitation, as neoadjuvant chemoradiotherapy patients are most likely to benefit from preoperative stratification. Future studies should focus on evaluating imaging biomarkers before chemoradiotherapy and correlating them with post- chemoradiotherapy outcomes. However, this will involve challenges such as the timing of imaging acquisition, variations in treatment regimens, and the need for longitudinal data. Fifth, certain histologic subtypes (*e.g*., mucinous adenocarcinoma) were excluded; broadening the case spectrum in future studies would improve generalizability. Finally, the moderate class imbalance in both the training and validation cohorts may have impacted the model’s stability and performance. While oversampling was applied to address this imbalance, it remains a potential limitation.

In conclusion, our study developed a combined model integrating histogram features of APTw and ADC images with clinical features. It demonstrated promising predictive performance and generalizability, which may enhance preoperative decision-making and support personalized therapeutic strategies.

## Supplementary information

**Additional file 1:** **Table S1.** Parameters of MR sequences. **Table S2.** ICCs of histogram parameters measured by 2 observers. **Table S3.** Optimal histogram features and standardized regression coefficients **Figure S1.** Boxplots of remarkable histogram features in the training cohort.

## Data Availability

Due to privacy restrictions, raw data cannot be made available free of charge, but datasets used and/or analyzed during the current study may be obtained from the corresponding authors upon reasonable request.

## Abbreviations

3D: Three-dimensional
ADC: Apparent diffusion coefficient
APTw: Amide proton transfer-weighted
AUROC: Area under the receiver operating characteristic curve
CA19-9: Carbohydrate antigen 19-9
CEA: Carcinoembryonic antigen
CI: Confidence interval
DWI: Diffusion-weighted imaging
ICC: Intraclass correlation coefficient
MRI: Magnetic resonance imaging
MRI-N: N stage at MRI
MRI-T: T stage at MRI
OR: Odds ratio
RC: Rectal cancer
SI: Signal intensity
T2WI: T2-weighted imaging
TB: Tumor budding
VOI: Volume of interest
