# Supplementary material for: Preoperative prediction of tumor budding grade in rectal cancer by combining APT histogram analysis and ADC MRI
Source: Eur Radiol Exp. 2026 May 20;10:68. doi: 10.1186/s41747-026-00727-w (PMC13190968; doi:10.1186/s41747-026-00727-w)
Supplement: Supplementary file 1 — Additional file 1: Table S1. Parameters of MR sequences. Table S2. ICCs of histogram parameters measured by 2 observers. Table S3. Optimal histogram features and standardized regression coefficients Figure S1. Boxplots of remarkable histogram features in the training cohort. [file 41747_2026_727_MOESM1_ESM.pdf]

Preoperative prediction of tumor budding grade in rectal cancer by combining APT histogram analysis and ADC MRI

ELECTRONIC SUPPLEMENTARY MATERIAL

Table S1. Parameters of MR sequences

Parameter	HR-T2WI	DWI	T2WI-tra	APTw
Repetition time/echo time (ms)	4030/90	3736/77	4592/100	6458/8.8
Section thickness （mm）	3.5	5	5	6
Field of view (mm)	200 × 200	350 × 298	300 × 377	280 × 260
Matrix	252 × 243	136 × 97	352 × 402	156 × 154
Flip angle (degree)	90	90	90	90
Spatial resolution (mm^3)	0.8 × 0.8 × 3.5	2.6 × 3 × 5	0.85 × 0.85 × 5	1.8 × 1.8 × 6
b-value (s/mm^2)	/	0.1000	/	/
Fat suppression	/	SPAIR	/	SPIR

APTw, Amide proton transfer–weighted; DWI, diffusion-weighted imaging; HR-T2WI, high-resolution T2-weighted imaging; T2WI-tra: Axial T2-Weighted Imaging; SPAIR, Spectral Attenuated Inversion Recovery; SPIR, Spectral Presaturation with Inversion Recovery.

Table S2. ICCs of histogram parameters measured by 2 observers

Histogram parameter	ICC	95% CI	*P*	Histogram parameter	ICC	95% CI	*P*
APTw-10%	0.83	0.64-0.93	<.05	ADC-10%	0.46	0.06-0.73	.01
APTw-90%	0.75	0.48-0.89	<.05	ADC-90%	0.91	0.79-0.96	<.05
APTw-Energy	0.86	0.69-0.94	<.05	ADC-Energy	0.70	0.41-0.87	<.05
APTw-Entropy	0.94	0.85-0.97	<.05	ADC-Entropy	0.77	0.51-0.90	<.05
APTw-IQR	0.93	0.45-088	<.05	ADC-IQR	0.75	0.50-0.89	<.05
APTw-Kurtosis	0.30	−0.13-0.63	.09	ADC-Kurtosis	0.84	0.65-0.93	<.05
APTw-Maximum	0.78	0.55-0.90	<.05	ADC-Maximum	0.75	0.50-0.89	<.05
APTw-MAD	0.65	0.32-0.84	<.05	ADC-MAD	0.66	0.34-0.84	<.05
APTw-Mean	0.94	0.85-0.97	<.05	ADC-Mean	0.84	0.65-0.93	<.05
APTw-Median	0.96	0.90-0.98	<.05	ADC-Median	0.81	0.60-0.92	<.05
APTw-Minimum	0.33	−0.10-0.66	.06	ADC-Minimum	0.80	0.60-0.91	<.05
APTw-Range	0.43	0.02-0.71	.02	ADC-Range	0.81	0.60-0.92	<.05
APTw-RMAD	0.70	0.40-0.86	<.05	ADC-RMAD	0.75	0.46-0.88	<.05
APTw-RMS	0.73	0.45-0.88	<.05	ADC-RMS	0.88	0.73-0.95	<.05
APTw-Skewness	0.47	0.07-0.74	.01	ADC-Skewness	0.77	0.52-0.90	<.05
APTwTotalEnergy	0.86	0.69-0.94	<.05	ADCTotalEnergy	0.62	0.27-0.82	.001
APTw-Uniformity	0.93	0.85-0.97	<.05	ADC-Uniformity	0.83	0.64-0.93	<.05
APTw-Variance	0.61	0.26-0.82	<.05	ADC-Variance	0.48	0.08-0.75	.01

ADC, Apparent diffusion coefficient; APTw, amide proton transfer–weighted; CI, confidence interval; ICC, intraclass correlation coefficient; IQR, Interquartile Range; MAD, mean absolute deviation; RMAD, robust MAD; RMS, root mean square.

Table S3. Optimal histogram features and standardized regression coefficients

Histogram feature	Coefficient
ADC_original_firstorder_90%	1.46053785242045
ADC_original_firstorder_Minimum	−0.871831543673189
ADC_original_firstorder_Range	−0.613529859000623
APTw_original_firstorder_10%	0.428792203845734
APTw_original_firstorder_ Median	0.602455724114956

ADC, Apparent diffusion coefficient; APTw, amide proton transfer–weighted.

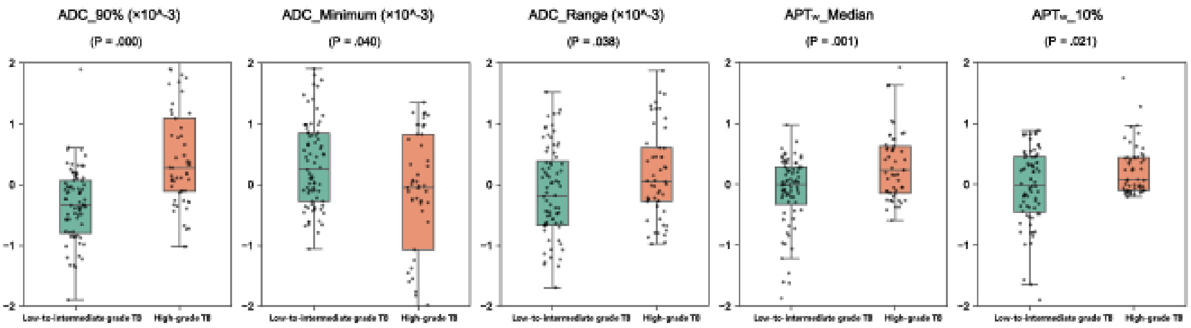

Supplementary Figure S1. Boxplots of remarkable histogram features in the training cohort. ADC, Apparent diffusion coefficient; APTw, amide proton transfer–weighted.

Supplementary File 2


```
featureClass:
  firstorder: []
setting:
  binWidth: 25
  force2D: false
  interpolator: sitkBSpline
  normalize: true
  normalizeScale: 100
  outlierLower: -500
  outlierUpper: 500
  removeOutliers: true
  resampleBySpacing: true
  resampledPixelSpacing: 1×1×1
  smooth: true
  wavelet: false
  waveletLevel: 3
```
